# Effect of Serenoa repens (Permixon®) on the expression of inflammation-related genes: analysis in primary cell cultures of human prostate carcinoma

**DOI:** 10.1186/1476-9255-10-11

**Published:** 2013-03-14

**Authors:** Ida Silvestri, Susanna Cattarino, AnnaMaria Aglianò, Chiara Nicolazzo, Susanna Scarpa, Stefano Salciccia, Luigi Frati, Vincenzo Gentile, Alessandro Sciarra

**Affiliations:** 1 Department of Molecular Medicine, Sapienza University of Rome, Rome, Italy; 2 Department of Urology, Sapienza University of Rome, Rome, Italy; 3 Department of Experimental Medicine, Sapienza University of Rome, Rome, Italy; 4 Department of Experimental Medicine and Pathology, Sapienza University of Rome, Rome, Italy; 5 Prostate Unit - Department Urology, University Sapienza, Viale Policlinico 155, 00161, Rome, Italy

**Keywords:** Apoptosis, Proliferation, Cell cultures, Inflammation, Prostate neoplasm, Caspase detection

## Abstract

**Background:**

To analyze the expression at basal level of inflammation-related cytokines and chemokines and the activation status of the NF-κB pathway, together with the proliferation and apoptosis indexes in two widely used in vitro tumor models, the androgen-dependent human Prostate Cancer (PC) cell line LNCaP and the androgen-independent PC3 , and in primary cultures of human PC cells. To assess in these models and primary cultures, the effects of *Serenoa repens* (LSESr, Permixon®) on proliferation/apoptosis ratio, inflammation-related genes expression and NF-κB pathway activation.

**Methods:**

The expression of IL-6, CCL-5, CCL-2, COX-1, COX-2, iNOS inflammation-related genes has been evaluated at the mRNA level in two in vitro human PC models (LNCaP and PC3 cell lines) and in 40 independent human prostatic primary cultures obtained from PC patients undergoing radical prostatectomy. Tissue fragments were collected from both PC lesions and normal hyperplastic tissue counterparts for each case. All cultures were treated with two different amounts of Permixon® (44 and 88 μg/ml) for different time points (16, 24, 48 and 72 hours), depending on the cell type and the assay; the expression of inflammation-related genes, cell growth (proliferation/apoptosis ratio) and NF-κB activation has been analyzed in treated and untreated cells by means of semi-quantitative RNA-PCR, cell proliferation and immunofluorescence respectively.

**Results:**

We detected a significant reduction (p <0.001) in PC and normal cells proliferation due to Permixon ® treatment. This result was related to an increase of the apoptotic activity showed by an increase in the number of anti-caspase-3 fluorescent cells. Almost all the inflammation-related genes (IL-6, CCL-5, CCL-2, COX-2 and iNOS) were expressed at the basal level in *in vitro* cultured cells and primary cultures and down-regulated by Permixon® treatment. This treatment interfered with NF-kB activation, detecting by the translocation of more than 30% of NF-κB p65 subunit to the nucleus.

**Conclusions:**

The present study confirms the expression of inflammatory pattern in PC. We showed the effect of Permixon® on down-regulation of inflammatory-related genes in cell lines and in primary cultures. The inhibitory effect of Permixon® on cell growth could be partly associated to the down-regulation of inflammatory-related genes and to the activation of NF-κB pathway in prostate tissue.

## Introduction

Evidences in literature suggest that prostatic inflammation is involved in the pathogenesis and progression of prostatic diseases, such as benign prostatic hyperplasia (BPH) and prostate cancer (PC). Epidemiologic, histopathologic and molecular pathologic studies support the association between prostate inflammation and prostate diseases, suggesting a possible mechanism of action [1-7].

The lipidosterolic extract of the fruits of the American dwarf palm tree *Serenoa repens* (LSESr) (Permixon) is probably the most studied phytotherapeutic drug, widely used for lower urinary tract symptoms (LUTS) treatment. A large number of pharmacodynamic effects, observed in vitro and in vivo, suggest multiple mechanisms of action on the human prostatic tissue, such as the anti-androgen effect [8,9], and the interference with mediators of inflammation [10-12] and with proliferative-apoptosis pathways [13]. Different studies [10-12], which tested the pharmacological properties of this drug on the inflammatory status of the prostatic tissue, concluded that the LSESr might have a potential anti-inflammatory effect.

The aim of our study is to analyze and to compare the expression of the inflammatory pathways in human PC cell lines, such as androgen-dependent LNCap and androgen-independent PC3 cell lines and in primary cultures of human prostate adenocarcinoma cells. On these different settings, we also evaluated the effect of LSESr (Permixon) on different inflammatory factors, analyzing whether its potential anti-inflammatory activity affects proliferation and apoptosis.

## Materials and methods

### Cell lines

LNCaP and PC3 cell lines were obtained from Interlab Cell Line Collection (ICLC) (Istituto Nazionale per la Ricerca sul Cancro, Genoa, Italy).

Human cancer epithelial cell line PC3 was grown in DMEM (Euroclone, Life Science Division, GB, Pero, Italy) supplemented with 10% fetal bovine serum (Euroclone) and LNCaP in RPMI 1640 (Euroclone). Cells were maintained in a tissue culture incubator at 37°C, 5% CO2. Cells were treated with LSESr (44–88 μg/ml) at different incubation times (24, 48 and 72 hours) and after RNA extraction, RT-PCR assay was performed at the same intervals of time.

### Ex vivo primary cultures

This experimental study was conducted after approval of the protocol from our Institutional Board Committee and informed consent was obtained from all patients. Exclusion criteria for the study were: previous hormonal, surgical or radiation therapies for prostate diseases; acute inflammatory diseases. Table 1 summarizes the characteristics of donors.

**Table 1:** Clinical characteristics of the 40 cases included for the analysis on primary cultures (number of cases and mean ± SD)

**Number of cases**	**40**
Age (years)	65.0 ± 3.3
Prostate volume (ml)	43.4 ± 6.8
PSA (ng/ml)	6.8 ± 2.4
Pathological stage	36 pT2
	4 pT3a
Pathological Gleason score	27 ≤ 7 (3 + 4)
	13 ≥ 7 (4 + 3)

We derived human epithelial cultures from tissue explanted from 40 patients undergoing radical prostatectomy for prostate adenocarcinoma. At surgery, after prostate removal, tissue samples were put on ice in Falcon tube containing 5 ml of minimum essential medium (Euroclone) and transferred in laboratory where they were immediately processed. From these cases (40 cases), we processed tissue fragments either from PC nodules (named Tumor “T”) or from normal tissue (named control “Ct”). The histological status of the tissue was checked by an independent pathologist.

In brief, the fresh tumor and normal tissue specimens were cut in small pieces and digested with trypsin under stirring 2 hours at 37°C. After filtration through 70 μm nylon cell strainer, epithelial cells were grown in PrEGM (Prostate Epithelial Growth Medium), containing the standard prostatic epithelial cell media additives (PrEGM Bullet Kit), including bovine pituitary extract, insulin, transferrin, epidermal growth factor, hydrocortisone, retinoic acid, epinephrine, and tri-iodothyronine, from Lonza.

After two weeks, when the different cells showed an exponential growth rate, they were treated with LSESr (Permixon®, Pierre Fabre Médicament, Castres, France).

### Preparation of LSESr (Permixon®) and stimulation settings

LSESr (Permixon) was obtained as a hexane extract from Pierre Fabre Medicament (Castres, France). The hexane was evaporated to leave the solid extract. The solid was dissolved in 10 ml of ethanol, to give a concentration of 10 mg/ml. This stock solution was further diluted in appropriate media to provide a working solution of 1 mg/ml.

### Cell counting

To determine the cell counting, LNCaP, PC3 and primary prostate cells were plated and subconfluent cells were treated with LSESr (Permixon) at a concentration of 44 and 88 μg/ml at different incubation times (6, 9, 24, 48 and 72 hours). After treatment cells were trypsinized, washed in-Phosphate buffered saline (PBS) and after centrifugation (5 min at 500 g) were resuspended in PSB (50-100 μl). An aliquot of each cell line was diluted 1:1 with 0.4% of trypsan blue. Cells were then loaded into a Neubauer chamber and living cells (unstained) were counted under a light microscope. Each assay was carried out in triplicate and the mean value was determined.

### Cyototoxic assay

LNCaP and PC3 cells were seeded in triplicate in 96-well plates at 5.0 × 104 cells per well. After 24 hours, *Serenoa Repens* (44 μg/ml) was added in a red phenol free DMEM medium for 48 and 72 hours, then cells were incubated with 2,3-bis[2-methoxy-4-nitro-5-sulfophenyl]-2H-tetrazolium-5-carboxanilide inner salt (XTT; Cell Proliferation kit Sigma-Aldrich) following the manufacturer’s instruction.

Absorbance was measured at 450 nm using a microplate reader (Labsystem Multiskan MS), the absorbance at 690 nm was subtracted from the 450 nm value and cytotoxicity was calculated by comparing absorbance of treated cultures with the absorbance of the untreated cultures at 48 and 72 hours.

### Caspase detection

In PC3 cell line and in primary cultures, anti-activated Caspase-3 antibody (Cell Signalling Technology) was used to carry out immunofluorescence assay and to investigate apoptosis induced in different cells by LSESr (Permixon). Cells were grown on Labteck chamber slides (Nunc, Naperville, Illinois, USA) for 24 hours, then treated with LSESr (Permixon) 44 μg/ml for 16 hours and analysed with immunocytochemistry using anti-cleaved caspase-3 and rhodamine indirect labelling. Cells treated and controls were fixed and permeabilized in methanol at −20°C for 10 min, rinsed in PBS, incubated for 1 hour at room temperature with TRITC-conjugated anti-rabbit IgG (1:500, Molecular Probes) and then examined under fluorescence microscope (Olympus BX-52).

### Reverse transcriptase polymerase chain reaction (RT-PCR) assay

Total RNA from LNCaP, PC3 and human prostate primary cells were extracted using Trizol reagent (Invitrogene, Carlsband, CA) according to the manufacturer’s instructions. High quality RNA preparations have been re-suspended in nuclease-free water and subjected to semi-quantitative polymerase chain reaction (RT-PCR). Moloney murine leukemia virus (M-MLV) reverse transcriptase (Biolab) was used to convert 1 μg of total RNA into cDNA at 42°C. 5 μg of each cDNA was then subjected to RT-PCR in a buffer containing 25 pmol of upstream and downstream and 1.25U of Platinum Taq polymerase (Euroclone). The amount of amplified products, expressed in arbitrary optical density units, was normalized with glyceraldehyde-3-phosphate dehydrogenase (GADPH) as housekeeping gene. The sequences of human gene-specific primers and the conditions of amplification as well as the amplified products size are listed in Table 1, with order of forward and reverse. The amplification reaction was carried out in PCR-expressed cyclers (TECHNE USI Instrument). The resulting PCR products were separated in 2% agarose gel and visualized with ethidium bromide. The primer sequences, amplification sizes and amplification conditions for each gene are listed in Table 2.

**Table 2:** Primers sequences and amplification conditions

**Gene**	**Sequences 5’-3’**	**Cicli**	**Annealing (temp C)**	**Size (bp)**
GADPH	ACATGTTCCAATATGATTCC	30	60°×30”	180
	TGGACTCCACGACGTACTCAG			
Il-6	CCTCCAGAACAGTTTGAGA	30	56°×1’	280
	CCTTAAAGCTGCGCAGAATG			
CCL-5	CTCGCTGTCATCCTCATTGCT	30	62°×30”	394
	TACTCCCGAACCCATTTCTTCTC			
CCL-2	ATG AAA GTC TCT GCC GCC CTT CTG T	36	60°×30”	286
	AGT CTT CGG AGT TTG GGT TTG CTT G			
COX-1	TGC CCA GCT CCT GGC CCG CCG CTT	30	60°×1’	304
	GTG CAT CAA CAC AGG CGC CTC TTC			
COX-2	TTC AAA TGA GAT TGT GGG AAA AT	30	60°×1’	305
	AGA TCA TCT CTG CCT GAG TAT CTT			
iNOS	TGG TGC TGT ATT TCC TTA CGA GGC GAA GAA GG	35	60°×45”	259
	GGT GCT TCT TGT TAG GAG GTC AAG TAA AGG GC			

The PCR amplification programme was as follows: an initial period of 5 min at 95°C, followed by a variable number of cycles of denaturation at 95°C for 30 sec, annealing (see Table 1) and finally 30 sec of extension at 72°C. The programme was terminated with a period of 10 min at 72°C. To be within the exponential phase of the semiquantitative PCR reaction, the appropriate number of cycles was newly established for every set of samples. PCR products were separated by gel electrophoresis on 15% agarose gels and visualized by ethidium bromide staining. All gels were scanned and the normalizedintensities of all reverse transcription (RT)-PCR products were determined by the BioRad gel documentation system (BioRad, Hercules, CA, U.S.A.). Mean ± SEM intensities were calculated for all RT-PCR experiments.

### Immunofluorescence

The protocol was performed only in LNCaP and PC3 culture cell lines. Cells were grown on Labteck chamber slides (Nunc, Naperville, Illinois, USA) and treated with LSESr (Permixon). After treatment, cells were washed with phosphate-buffered saline (PBS) and fixed with absolute methanol for 5 minutes at −20°C. Cells were then incubated for 1 hour with rabbit polyclonal antibody to p65 (Santa Cruz Biotechnology), rinsed three times with PBS and then incubated with fluorescein isothiocyanate-conjugated anti-rabbit IgG (Sigma, Milano, Italy) for 1 hour. Cells were then rinsed three times with PBS and mounted with Prolong anti-fade reagent, and the fluorescence was analyzed by an Olympus BX52 (Hamburg, Germany) fluorescence microscope. The images were acquired and elaborated with IAS 2000 software (Delta Sistemi, Rome, Italy).

### Statistical analysis

Statistical significance was determined using a one tailed Student’s *t* test (PRISM statistical analysis software) and data were expressed as means ± standard deviation (SD) of independent samplings from different experiments. A cut-off of p < 0.05 was used to demonstrate significance.

## Results

### Cell counting, citotoxicity assay and apoptosis activation

**Figure 1:**
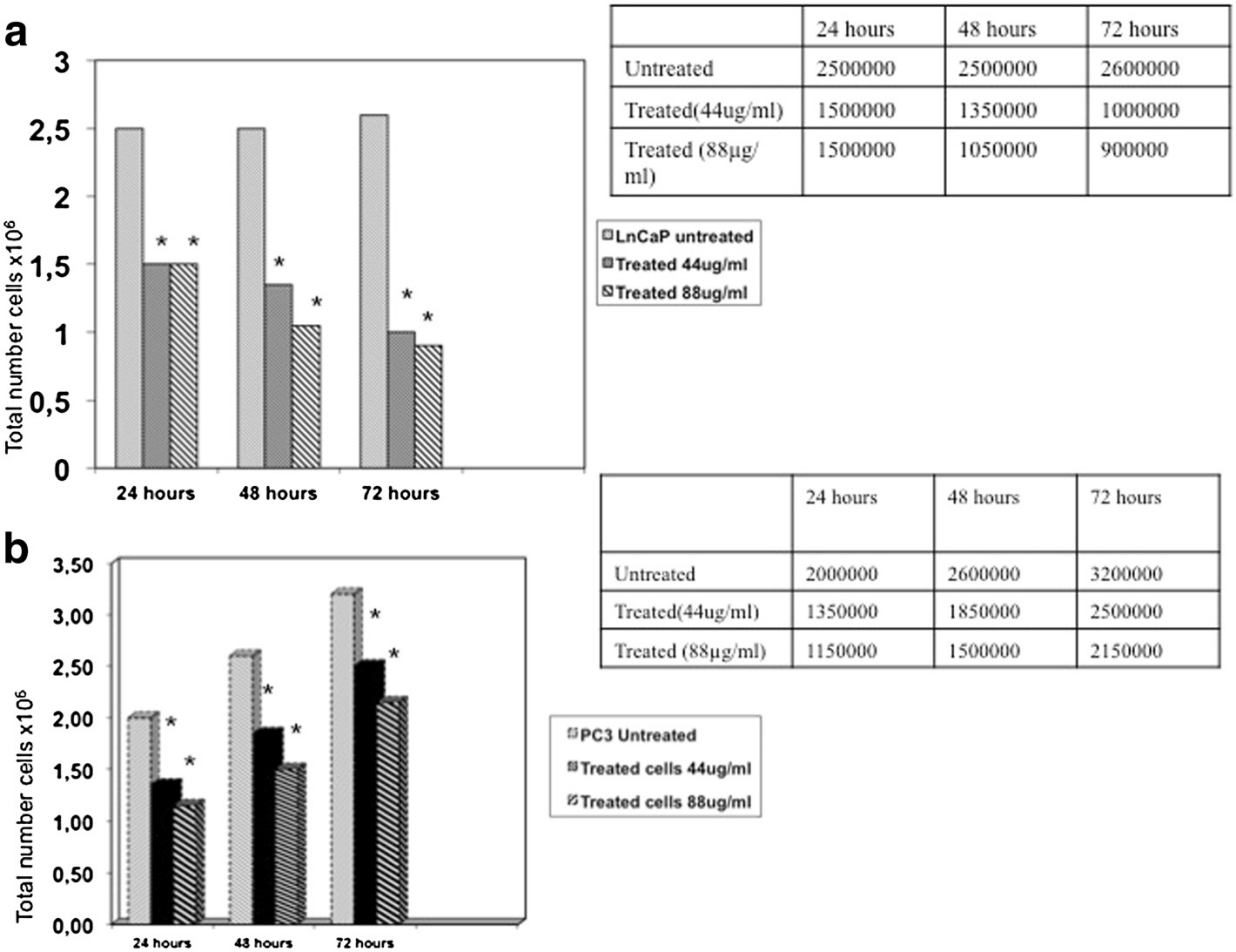
**Cell counting by trypsan blue in LNCaP and PC3 cell lines.** Results are presented at 24, 48 and 72 hours of incubation, either in untreated or in Permixon® treated (44 and 88 μg/ml) conditions. Cells were seeded at different concentration, lower for longer incubation time in order to avoid overgrowth. The same amount of cells was used to start each incubation time, control and treated cells. **a)** LNCaP. **b)** PC3. * = p < 0.05.

**Figure 2:**
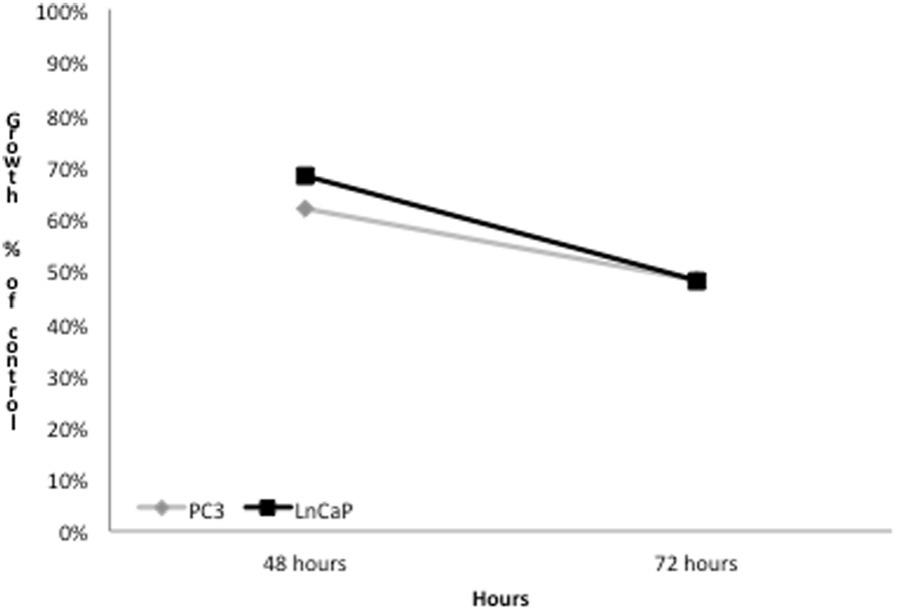
**Cytitoxicity assay in LNCaP cell and PC3 lines.** Results are presented at 48 and 72 hours of incubation, either in untreated or in Permixon treated (44 and 88 μg/ml) conditions. The graphic shows the reduction of cell growth according to the absorbance of treated and untreated cultures (XTT Cell Proliferation kit Sigma-Aldrich).

To evaluate the inhibitory effect of Permixon® on in vitro cell growth, we analyzed cell viability by direct cell counting. As shown in Figure 1, in both LNCaP and PC3 tumor cell lines, Permixon® treatment significantly reduced cell growth at all time points (24, 48 and 72 hours after stimulation) when compared to untreated cells (p < 0.05). Reported effects are not dose-dependent as differences achieved with the two doses of Permixon® (i.e. 44 and 88 μg/ml) were not statistically significant (p > 0.05). A similar result was observed also using XTT (Figure 2).

The cells of 90% out of 40 independent human prostate primary specimens were grown in ex-vivo cultures. Human samples were screened by means of RT-PCR for the expression of CD45 and CK5/CK8 markers to assess the epithelial origin of cultured cells; only CD45 negative/CK5-CK8 positive cultures have been used for in vitro assays, although little contamination could not be completely excluded (36).

**Figure 3:**
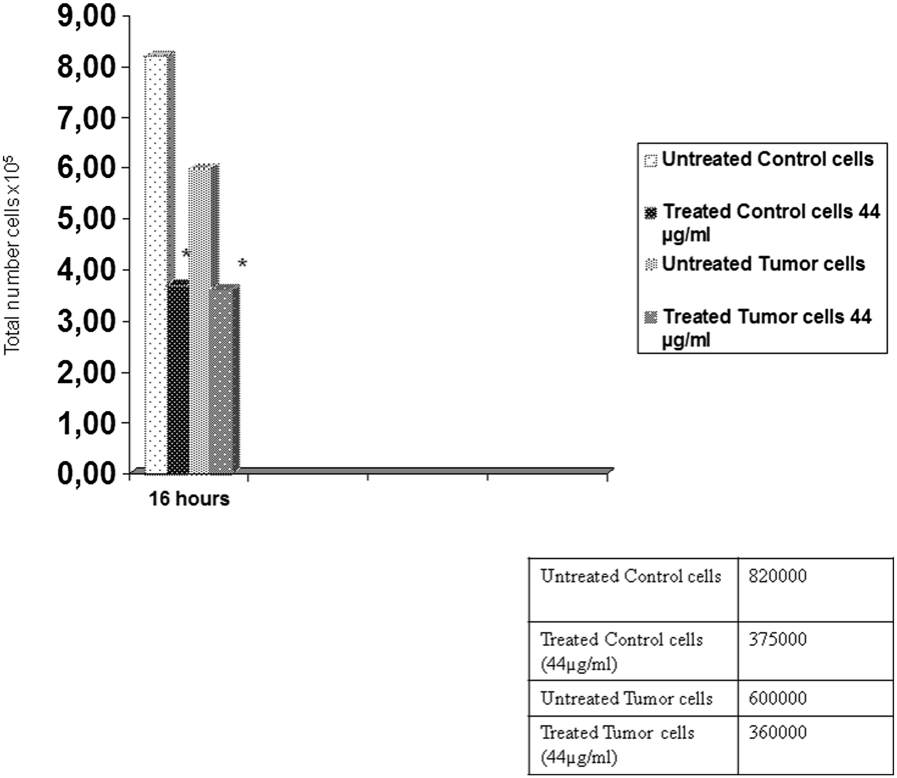
**Cell counting in human primary cells.** Results are presented at 16 hours of incubation either in untreated or in Permixon® treated (44 μg/ml) conditions. This result represents the total number of cells harvested after 16 hours of incubation. The same amount of cell was seeded for each control and treated cells. Tumor cells = prostate adenocarcinoma primary cultures; Control cells = normal cell primary culture. * = p < 0.05.

Cultured human prostate primary cells were in vitro expanded and assayed for cell growth. A relatively slow cell proliferation rate has been observed in all human samples and none of the ex-vivo cultures has been immortalized so far. Furthermore, cell growth of human primary cells was more promptly inhibited by Permixon® compared to LNCaP and PC3 cell lines. Consequently, on human primary samples we reported the effect of only one dose of Permixon® (44 μg/ml) and cells have been collected after 16 hours of stimulation. As shown in Figure 3, both in the tumoral and normal primary counterparts, Permixon® reduced significantly cell growth when compared to untreated controls (p < 0.05).

**Figure 4:**
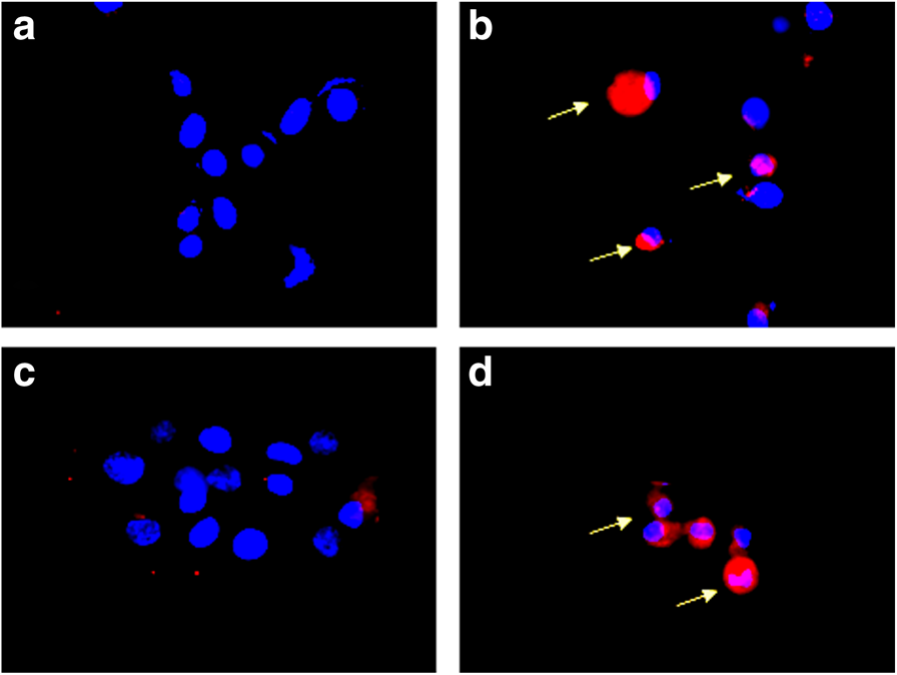
**Detection of caspase-3 in apoptotic cells.** Immunocytochemistry (immunofluorescence) with anti-cleaved caspase3 by rhodamine indirect labelling. The upper panels are PC3 untreated (**a**) and treated (**b**) with LSESr for 16 hours. The lower panels are primary cells line untreated (**c**) and treated (**d**) with Permixon® for 16 hours. Untreated cells show no labelling with the anti-cleaved caspase-3 antibody and TRITC anti IgG. LSERSr treated cells show (arrows) labelling with anti-cleaved caspase-3.

To determine whether Permixon® induced inhibition of cell growth was due to an increase in apoptosis index, the caspase-3 activation has been evaluated by means of immunofluorescence. This assay has been performed on PC3 cell line and human primary cultures. Results showed a significant (p > 0.05) increase in the number of detached cells (mainly in human primary samples) and of anti-caspase-3 fluorescent cells (Figure 4).

### Inflammation-related genes expression

To evaluate in all our experimental samples the expression pattern of inflammation-related genes in untreated (in vitro basal level) and Permixon® treated conditions, we performed expression analysis at RNA level of the following genes: IL-6, CCL-5 (RANTES), CCL-2, COX-1, COX-2 and iNOS.

#### IL-6

**Figure 5:**
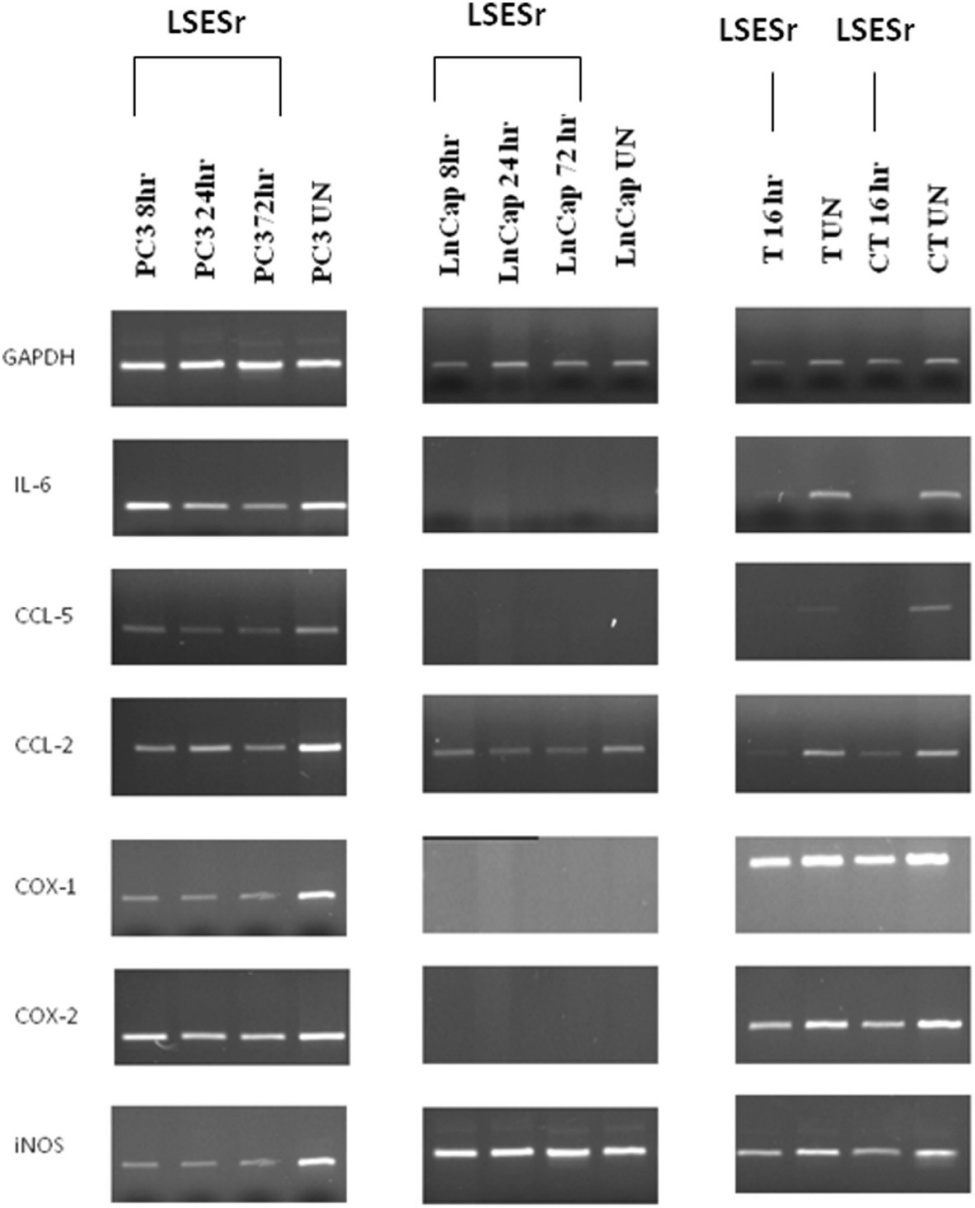
**GADPH, IL-6, CCL-5 (RANTES), CCL-2, COX-1, COX-2 and iNOS RT-PCR products separated on a 2% agarose gel followed by ethidium bromide staining in PC3, LNCaP and primary cells lines.** In PC3 (panel 1) and LNCaP (panel 2) analysis was performed at 8, 24 and 72 hours of incubation whereas in primary cell lines (panel 3) at 16 hours of interval. Here we report results obtained from one prostatectomy, representative of 40 independent experiments. The experiment was performed in untreated and LSESr treated (44 μg/ml and 88 μg/ml in PC3 and LNCaP and only 44 μg/ml in primary cultures) conditions. UN = untreated; LS = LSESr treated; T = primary cells culture derived from PC nodules; Ct = primary cells culture derived from normal prostate tissue surrounding the tumor.

In untreated conditions we observed elevated expression of IL-6 in PC3 cells, while no expression was detected in LNCaP cells. In untreated in vitro cultured human primary cells we detected a high expression of IL-6, although lower if compared to the expression in PC3 cells. In both PC3 cells and human primary cells, Permixon® treatment induced a down-regulation of IL-6 gene, (mainly at 24 and 72 hours in PC3 cells and even after 8 hours in human primary cells), Figure 5.

#### CCL-5 (RANTES)

In untreated conditions we observed high expression of CCL-5 gene in PC3 cells. As for IL-6 gene, also for CCL-5, we could not detect expression in LNCaP cells. Permixon® treatment, at all time points analyzed (8, 24 and 72 hours) reduced CCL-5 expression in PC3 cells. The expression of CCL-5 in untreated human primary cells was higher compared to PC3 cells and even after 8 hours of Permixon® treatment, we observed a down-regulation of this gene in primary cells, Figure 5.

#### CCL-2

We observed high expression of CCL-2 in both PC3 and LNCaP untreated cell lines and reported gene down-regulation after 8, 24 and 72 hours of in vitro Permixon® treatment. A similar high level of expression has been observed in human primary cultures and similar trend of down-regulation after 16 hours of Permixon® treatment, Figure 5.

#### Cyclo-oxygenase 1 and 2 (COX-1 and COX-2)

We observed elevated levels of COX-1 expression in PC3 untreated cells while no expression was detected in LNCaP untreated cells. Permixon® treatment had significant effect on the expression of COX-1 gene in treated PC3 cells. Primary cell cultures, from both tumor and normal tissue specimens, showed high level of COX-1 expression, but on these samples Permixon® treatment had no effects (Figure 5). On the other hand, COX-2 expression was high in untreated PC3 cells and human primary cultures, while no expression was detected in untreated LNCaP cells. Permixon® treatment, at all time points analysed (8, 16, 24 and 72 hours), did not show down-modulation of COX-2 gene expression.

#### Inducible Nitric Oxide Synthase (iNOS)

Although high level of iNOS expression has been detected in both PC3 and LNCaP cell lines, Permixon® treatment had effect on gene down-modulation only in PC3 cells, while no effect has been detected in LNCaP cells. In human primary cells, Permixon® reduced iNOS gene expression even after 16 hours of treatment, Figure 5.

### NF-κB pathway activation

**Figure 6:**
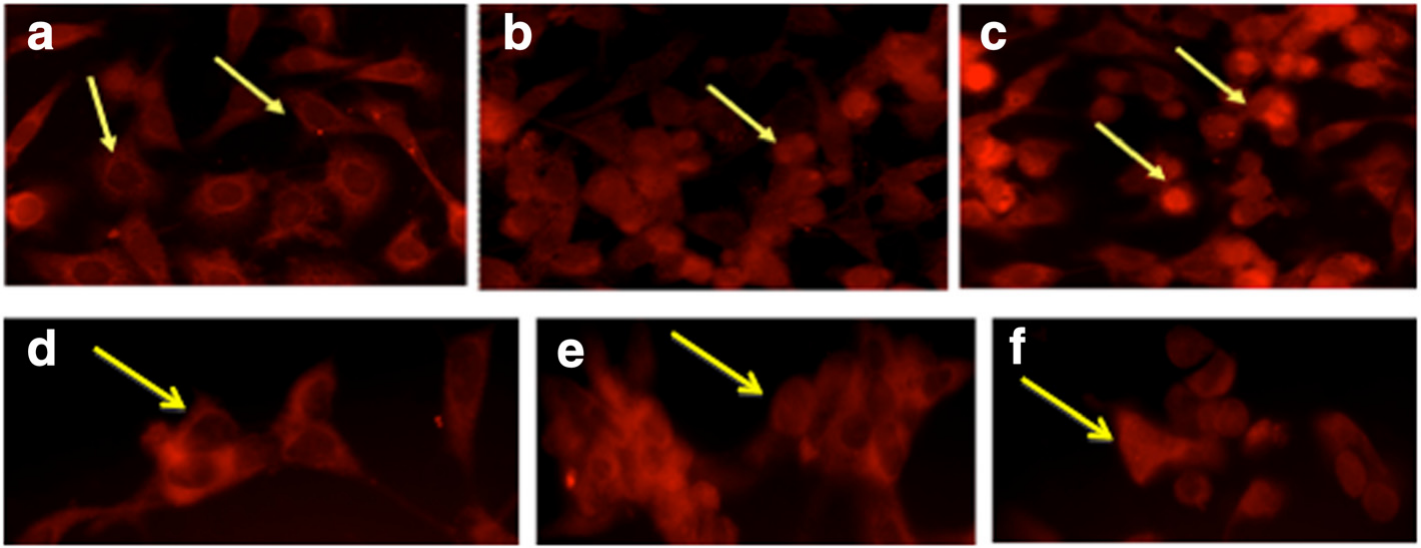
**NF-κB detection.** Immunofluorescence of p65 NF-κB in PC3 (**a, b, c**) and LNCaP (**d, e, f**) cell lines. In untreated condition (**a, d**) NF-κB is 100% detected in the cytoplasm of cells (arrows). After LSESr (44 μg/ml) treatment (**b, e**) = 24 hours and (**c, f**) = 48 hours of incubation more than 30% of NF-κB translocated at nuclear level (arrows).

Analysis of NF-κB activation has been performed only on LNCaP and PC3 cell lines. In untreated conditions NF-κB p65 subunit was detected only in the cytoplasm, while 24 and 48 hours after Permixon® treatment more than 30% of NF-κB p65 subunit translocated to the nucleus, Figure 6. Therefore LSESr treatment can also interfere with NF-kB activation.

## Discussion

This study shows for first time that primary human PC cells are influenced in growth and apoptosis pathways by natural compounds such as LSESr (Permixon), which down-regulates the inflammation pattern.

The use of primary prostatic tissue represent a good model of study for these experiments compared to cells in continue culture (LNCaP and PC3) because it reflects a more realistic biological state of human prostate cells [14,15]. It is considered that primary cells retain many phenotypic characteristics of the original tissue, including physiological functions and therefore they can be an appropriate model also for drugs testing.

Doses of LSESr (Permixon) and incubation times were sourced from the literature in PC cell lines, also if a large variability exists [16-19]. Regarding the dose of LSESr (Permixon), different studies suggested that 10 μg/ml well represents the calculated plasma concentration in patients receiving the recommended therapeutic dosage [18]. Previous studies showed that in PC cell lines higher doses are needed to obtain significant results, which are also influenced by a dose dependent effect [16-20].

The first result obtained in our study is that in every experiment (LNCaP, PC3 and primary cultures) we detected a significant reduction in PC cells counting caused by LSESr (Permixon) treatment. Comparable results were also observed in cells derived from normal prostate tissue. We stopped the analysis at 16 hours and used only 44 μg/ml of LSESr (Permixon) because the cells derived from primary cultures showed a more inhibited growth when incubated with LSESr (Permixon). These findings suggest that the sensibility to LSESr (Permixon) depends on the cell line, and the response is stronger in primary cultures.

Our results highlight that the inhibited growth observed after treatment with LSESr (Permixon) is determined by the activation of a cellular mechanism related to apoptosis. In the same condition, we also detected an induction of the apoptotic pathway, under LSESr treatment, caused by the caspase-3 activation [21,22].

However other studies detected apoptosis after *Serenoa repens* treatment [17,23]; some authors demonstrated that Permixon can effect prostate cancer cell growth without inducing apoptosis [24]. Different results are possibly due to: different preparation of the drug in various laboratories can contain a different active amount of *Serenoa repens* or same cell line after a long time in culture can get some different biological activity for mutation in its genotype [25].

The second result obtained from our study is that LSESr (Pemixon) produces a down-regulation of most of the inflammatory-related genes tested. We underline that this study analysed also COX and iNOS genes expression and we showed a down-regulation of both COX-1 and iNOS induced by LSESr (Permixon) in PC3 cell line as in several primary cell cultures.

In this study we had the possibility to evaluate in the same experiment and in primary cultures the major inflammatory-related genes, which might be responsible for influencing PC disease. All these inflammatory-related genes were expressed and all, except the COX-2 gene, were down-regulated by LSESr (Permixon) in primary cultures and in PC3 cell lines. However, in LNCap cell line, only CCL-2 and iNOS genes were expressed. The lack of expression for most of the inflammatory genes in LNCaP line may be related to the different origin of these cells, which are androgen-dependent and do not express IL-6 protein [26]. A quantitative analysis of inflammatory markers and their modifications under treatment should be very important and should be added at our experiments; this represent a strong limit of this study and other results are still necessary.

Finally, we analysed NF-kB gene expression by immunofluorescence. NF-kB is anchorated in the cytoplasm in an inactive form by the association with a cell family inhibitor of kappa B (IkB). Different signals can induce IkB phpsphorilation and degradation by proteosomes, so that NF-kB is translocated in the nucleus, where it can induce the transcription of multiple genes. Such NF-kB activation can induce either pathways that promote cell survival or other pathways that promote cell death. The different effects mediated by NF-kB depend on the activated signals and the type of tissue or cell line [27-31]. The antibody to NF-kB used in the present paper has been previously utilized by other authors to asses NF-kB activation by immunofluorescent staining of NF-kB nuclear translocation [32,33].

We showed that in LNCaP and PC3 line, LSESr (Permixon) induces activation of NF-kB through its translocation in the cell nucleus. A down-regulation of the different inflammatory-related genes and a significant reduction in PC cell counting related to an apoptotic process was found at the same time of incubation and concentration of LSESr (Permixon) in which NF-kB was activated.

Our findings support the hypothesis that LSESr (Permixon) might have an inhibitory effect on the expression of inflammation related genes. We can suppose that LSESr (Permixon) can stimulate NF-kB pathway leading to a pro-death response. However, we can expect that LSESr (Permixon) acts via different mechanisms to inhibit cell proliferation in LNCaP cell line. Nevertheless, the correlation of NF-kB with proliferation and apoptotic responses need further investigation caused by different androgen dependence and inflammation related gene expression.

## Conclusions

Our study contributes to a better understanding of the effect of inflammatory-related genes on PC cell growth and the potential inhibitory effect of LSESr (Permixon) on these pathways. In particular, using primary cultures from human PC tissue, we showed that the inhibitory effect of LSESr (Permixon) on cell growth could be in part associated to the down-regulation of inflammatory-related genes and to the activation of NF-kB pathway in prostate tissue.

## Competing interests

No potential conflict of interest was disclosed.

## Author’s contribution

IS carried out PCR analysis, organization of experimental evaluation on primary cultures. SC clinical evaluation of patients, surgical procedures, collecting data; manuscript writing. AMA carried out NFkB analysis by immunofluorescence. CN carried out cell count in primary cultures. SS carried out apoptosis experiments. SS clinical evalutation of patients, surgical procedures. LF final supervision and evaluation. VG supervisor; clinical management of patients. AS study ideation and management of patients; surgical procedure. All authors read and approved the final manuscript.
